# Reduced CHMP7 Expression Compromises Telomere Integrity in Mammalian Cells

**DOI:** 10.3390/cells15030256

**Published:** 2026-01-28

**Authors:** Romina Burla, Mattia La Torre, Klizia Maccaroni, Stefano Tacconi, Marco Fidaleo, Luciana Dini, Isabella Saggio

**Affiliations:** 1 Department of Biology and Biotechnologies “Charles Darwin”, Sapienza University of Rome, 00185 Rome, Italy; klizia.maccaroni@ifo.it (K.M.); stefano.tacconi@uniroma1.it (S.T.); marco.fidaleo@uniroma1.it (M.F.); luciana.dini@uniroma1.it (L.D.); 2 Institute of Molecular Biology and Pathology, CNR Rome, 00185 Rome, Italy

**Keywords:** telomeres, nuclear membrane, cancer, aging

## Abstract

**Highlights:**

**What are the main findings?**

- The reduction in the nuclear ESCRT CHMP7 drives telomeric aberrations.
- The reduction in the nuclear-envelope-assembly-related factors LEM2 and BAF1 drives telomeric aberrations.

**What are the implications of the main findings?**

- The ESCRT machinery is interlinked with telomere organization.
- Fragilization of telomeres can be an effect of mutations in ESCRT-encoding genes.

**Abstract:**

During open mitosis, reassembly of the nuclear envelope requires the coordinated recruitment of the ESCRT machinery, initiated by the chromatin-associated factor BAF1 and the nuclear-envelope-associated factor LEM2. Because telomeres are enriched at the reforming envelope, we investigated whether ESCRT factors contribute to telomere integrity. Reduction in the pivotal nuclear ESCRT factor CHMP7 caused DNA damage, heterochromatin disorganization, and telomere defects, including sister telomere associations and telomere free ends. Extending this analysis, we found that additional ESCRT components, including TSG101, VPS28, CHMP4B, and the ESCRT-associated factor AKTIP/Ft1, also contribute to telomere integrity, although with different strengths. Genetic interaction analyses suggest that CHMP7 converges in a common pathway with CHMP4B and AKTIP/Ft1, while it functions in parallel routes to TNKS1, a telomere-specific regulator of the shelterin TRF1. More genetic analyses indicated that BAF1 and LEM2 contribute to safeguarding of telomeres during nuclear envelope reassembly. Because defects in nuclear envelope dynamics and chromatin–membrane coupling are hallmarks of disorders associated with nuclear deformation and fragility, including aging and cancer, our findings contribute a new angle into these conditions and suggest potential targets for selectively modulating telomere maintenance pathways.

## 1. Introduction

Telomeres are specialized nucleoprotein structures located at the ends of linear chromosomes. Their main functions are protecting chromosomal ends from being recognized as DNA damage breaks and ensuring proper replication and lengthening [1,2]. Telomeres are formed by repetitive DNA sequences, which, in humans, are TTAGGG repeats. These DNA sequences are bound by specialized proteins, the shelterins. Shelterins include TRF1, TRF2, TIN2, TPP1, RAP1, and POT1. TRF1 and TRF2 bind to double-stranded telomeric repeats, while POT1 binds to single-stranded telomeric DNA. TRF1 and TRF2 are connected to POT1 via TPP1. Shelterins recruit to telomeres other factors that are needed for telomere functionality and maintenance. TRF1 interacts, for example, with tankyrase 1, a positive regulator of telomere length, required for sister telomere separation [3,4,5,6]. Telomere deprotection, caused either by their shortening beyond a critical length or by defects in genes encoding for telomeric proteins, generates their aberrant processing, cell cycle arrest, apoptosis or senescence, and genome instability [7,8].

Multiple pieces of evidence establish a link between telomeres and the nuclear periphery. This is particularly evident in fission yeast. Here, in interphase, telomeres form clusters at the nuclear envelope. It has been demonstrated that such positioning impacts on the stability of the subtelomeric chromatin and on the overall organization of the genome, affecting transcription and replication [9,10,11,12].

In mammalian cells telomeres are dispersed in the nucleoplasm in interphase [13,14,15,16]. However, in the final stages of mitosis and in early post-mitotic nuclei, telomeres are enriched at the nuclear periphery [17,18]. Proof of the connection between the nuclear envelope and telomeres in mammalian cells has been based on data obtained in diseased cells, including cells with mutations in the components of the nuclear lamina, and in aged and cancer-invading cells [19,20,21]. Additionally, early work on the shelterin TRF1 highlighted its association with the nuclear matrix [22].

At the anaphase stage, during the process of reassembly of the nuclear envelope, the enrichment of telomeres at the nuclear envelope of mammalian cells is observed. Indeed, at this stage, telomeres associate with factors that accumulate around the chromatin disks, such as the lamin-associated protein LAP2alpha and the non-specific chromatin binding factor BAF1 [18,23,24]. These latter factors, together with lamins and other lamin-associated factors, such as LEM2, are involved in the subsequent stages of nuclear envelope sealing and spindle fiber disassembly.

These latter processes depend on the activity of the ESCRT (Endosomal Sorting Complex Required for Transport) machinery. This is a membrane remodeling multimeric complex. Beyond controlling nuclear envelope reassembly and spindle disassembly, the ESCRT machinery contributes to nuclear envelope repair of interphase nuclei in case of breaks [25,26,27]. Additionally, the ESCRT machinery is involved in endosomal trafficking and in the final stage of cell division named abscission [28,29,30].

The ESCRT machinery is typically formed by the differential assemblage of ESCRTs type 0, I, II, and III. At the nuclear envelope work the ESCRTs type III CHMP2A, CHMP4B, and IST1 (also named CHMP8), which recruit the downstream ESCRT-associated ATPases VPS4 and spastin. These latter factors disassemble the ESCRT polymers and the spindle fibers. CHMP7 is another major ESCRT player at the nuclear envelope. It is an ESCRT hybrid type II/III and contributes to the aggregation of the ESCRTs CHMP4B, CHMP2A, and IST1 during nuclear envelope reassembly [28,29,31,32].

In this work, we considered the role of the ESCRT complex and, in particular, of CHMP7 in telomere maintenance. We report that the reduced expression of CHMP7 generates DNA damage foci and alters the organization of heterochromatin. Moreover, we show that CHMP7 is needed for telomere integrity since the reduction in its expression induces the formation of aberrant telomeres. We also show that not only the reduction in the ESCRT CHMP7, but also that of CHMP4B, LEM2, and BAF1 generate telomere aberrations. On the other hand, the reduction in IST1 does not. Co-reduction analyses suggest a genetic interaction of CHMP7 with other ESCRT factors and with the LEM2 and BAF1.

Altogether this study highlights a role for the ESCRT machinery, and for CHMP7 in particular, in telomere integrity. The study contributes to adding evidence supporting the link in mammalian cells between telomeres and the nuclear envelope.

## 2. Materials and Methods

### 2.1. Cells and Vectors

HeLa cells (ATCC CCL-2) and 293T cells (ATCC CRL-11268) were cultured in Dulbecco’s modified Eagle’s media (DMEM, GIBCO, ThermoFisher Scientific, Paisley, Scotland, UK) supplemented with 10% Fetal Bovine Serum (FBS, GIBCO, ThermoFisher Scientific, Paisley, Scotland, UK) and 1% (*v*/*v*) PenStrep. p53ko mouse embryonic fibroblasts were obtained as previously described [33,34] and cultured in the same medium. Unless otherwise specified p53ko mouse embryonic fibroblasts were derived by the mouse line described in [34]. The vector pMGF182, pCMV(Δ5)-GFP-CHMP7 (GFP-CHMP7) was a gift from Adam Frost & Wesley Sundquist (Addgene plasmid # 97006; http://n2t.net/addgene:97006, accessed on 2 January 2026; RRID:Addgene_97006) [35]. The vector YFP-TRF1 was derived from the eGFP- pWzl-hygro expressing vector [36], and HeLa cells stably expressing the YFP-TRF1 vector were a gift from A. Chojnowski and Oliver Dreesen (A*SRL, Agency for Science, Technology and Research (A*STAR), Singapore 138648 Singapore). All cells were grown at 37 °C and 5% CO_2_.

Stably silenced cell lines were obtained by lentiviral transduction. Lentiviral vectors were produced and titrated as previously described [37]. For human shCHMP7, murine shChmp7, murine shBaf1, murine shLem2, murine shChmp4b, murine shTnks1, and murine shFt1, the transfer vectors used were, respectively, TRCN0000150831, TRCN0000105450, TRCN0000124955, TRCN0000192997, TRCN0000105503, TRCN0000111416 and TRCN0000037370, TRCN0000244484, TRCN0000054606, TRCN0000266805 (Merck, Darmstadt, Germany). Untargeted shRNA, SHC002 (Merck, Darmstadt, Germany), was used as control (ctr). Cells were transduced with a multiplicity of infection of 3pg24/cell, in complete medium supplemented with polybrene 8 µg/mL (Merck, Darmstadt, Germany). After viral addition cells were centrifuged for 30 min at RT. Media were changed 3 h post viral addition. Seventy-two hours post-transduction, puromycin 2 µg/mL was added to complete medium and cells were kept in these conditions for further analyses. Unless otherwise specified, transduced cells were used for a maximum of 6 passages after puromycin selection.

Cell proliferation was monitored by cumulative population doubling and by the analysis of mitotic index. The population doubling was calculated with the formula Log(n_t_/n_0_) × 3.33, where n_0_ is the number of cells plated and n_t_ the number of cells after n passages. For the mitotic index, cells were plated on coverslips 2 passages after puromycin selection and after 48 h they were fixed with 3.7% Formaldehyde (Merck, Darmstadt, Germany) for 10 min at RT. DNA was stained with DAPI and slides were mounted with VECTASHIELD H- 1000. The mitotic index was calculated as the ratio of mitotic cells to the total number of cells in a field.

Rescue experiments were performed in control (ctr) and CHMP7-reduced (shCHMP7) HeLa cells obtained as described in this section. For GFP-CHMP7 expression, cells at passage 3 after puromycin selection were seeded 24 h before transfection and transfected at approximately 60% confluence. Antibiotic selection was removed 3 h before transfection. Cells were transfected following the manufacturer’s instructions with 8 µg of GFP-CHMP7 vector using Lipofectamine 2000 (Invitrogen by Thermo Fisher Scientific, Carlsbad, CA, USA) at a 1:2.5 DNA:Lipofectamine ratio. At 4 h after transfection, the transfection mixture was removed and replaced with fresh complete medium. Western blotting and lobular nuclear protrusion analyses were performed 48 h post-transfection and described in the following sections.

### 2.2. Gene Expression

For RNA expression, starting from 48 h of puromycin selection, RNA was extracted from transduced cells using Trizol (Life Technologies by Thermo Fisher Scientific, Carlsbad, CA, USA) following manufacturer’s instructions. cDNA was obtained by Quantitech Reverse Transcription Kit (Qiagen, Hilden, Germany). QPCR was performed as previously described [38] with SYBR Green Mix (QuantiTect SYBR Green PCR Kit, Qiagen Hilden, Germany) and using QuantStudio 3 (Applied Biosystem, ThermoFisher Scientific, Waltham, MA, USA).

The primers used are listed below:

hGAPDH Forward 5′-TGGGCTACACTGAGCACCAG-3′;
hGAPDH Reverse 5′-GGGTGTCGCTGTTGAAGTCA-3′;
hCHMP7 Forward 5′-AACTCTCCATGAAGGATGTC-3′
hCHMP7 Reverse 5′-ATCTAAGCCATTTGTTACCC-3′
mGapdh Forward 5′-GTGGCAAAGTGGAGATTGTTGCC-3′
mGapdh Reverse 5′-TGTGCCGTTGAATTTGCCGT-3′
mChmp7 Forward 5′-ATACCAGAACTCGCCCCTCT-3′
mChmp7 Reverse 5′-CAGCACGAGGTAGAAGGTCC-3′
mBaf Forward 5′-CTTTGACAAAGGCTTATGTGG-3′
mBaf Reverse 5′-CATGTATCCTTCAGCCATTC-3′
mLem2 Forward 5′-GACTGTGAGAGAAAAACAGATG-3′
mLem2 Reverse 5′-CACATTAGCTATGTACTCCTG-3′
mFt1 Forward 5′-CCGTCTTTCACCCACTAGTTGAT-3′
mFt1 Reverse 5′-TTGCGAACGCTCTTTTCACA-3′
mTnks1 Forward 5′-GAAAGACAAAGGTGGACTTG-3′
mTnks1 Reverse 5′-GTGACTAAGTAACAGAGAACAG-3′
mChmp4b Forward 5′-CATGGACATTGATAAGGTGG-3′
mChmp4b Reverse 5′-CCACTGATCTCCAACCAAATTC-3′
mTsg101 F 5′-CACATACCCATATAACCCCC-3′
mTsg101 R 5′-CTCCTCTCCAAATATCACAATC-3′
mVps28 F 5′-CTATGGAGAGGATCAAAGAGG-3′
mVps28 R 5′-GACTGTAATGAAGAGCGAAAC-3′
mIst1 F 5′-GGAAGTCGGCTTCTTCCGTT-3′
mIst1 R 5′-CTCGTAAGCGCTCAGCTTTG-3′

QPCR reactions were analyzed with the 2^−ΔΔCq^ method as previously described [37,39].

### 2.3. Western Blotting

Controls (ctr) and CHMP7-reduced (shCHMP7) HeLa cells were subjected to Western blotting analysis at passage 3 after puromycin selection. Controls (ctr) and stably Chmp7-reduced (shChmp7) p53ko mouse embryonic fibroblasts were subjected to Western blotting analysis at passage 4 after puromycin selection. Rescue experiment was performed on controls (ctr), and CHMP7-reduced (shCHMP7) HeLa cells at passage 3 after puromycin selection. For protein quantification, cell extracts were obtained as previously described [40]. Briefly, cells were lysed in 20 mM Tris-HCl, pH7.5, 1% SDS, 1 mM Na_3_VO_4_, 1 mM PMSF, 5% β-mercaptoethanol, and subjected to sonication at 14% amplitude for 30 s on ice. Samples were then centrifuged at 13,000 rpm at 4 °C for 30 min. Protein extracts were quantified with NANODROP Lite (Thermo Fisher Scientific, Waltham, MA, USA) and loaded onto pre-cast 4–12% gradient acrylamide gels (Nu-Page, Life Technologies by Thermo Fisher Scientific, Carlsbad, CA, USA). After electro-blotting, filters were incubated with anti CHMP7 (Proteintech, Rosemont, IL, USA, 16424-1 AP) and anti actin-HRP conjugated (Santa Cruz Biotechnology, Santa Cruz, CA, USA, sc-1615) antibodies. Filters were then incubated with appropriate HRP-conjugated secondary antibody (Santa Cruz Biotechnology, Santa Cruz, CA, USA, sc-2357). Detection was performed using the enhanced chemiluminescence system (SuperSignal™ West Femto Maximum Sensitivity Substrate, Life Technologies by Thermo Fisher Scientific, Carlsbad, CA, USA).

### 2.4. Transmission Electron Microscopy

Cells were fixed for one hour in 2.5% glutaraldehyde prepared in 0.1 M cacodylate buffer (pH 7.4), followed by a two-hour post-fixation on ice in 1% osmium tetroxide in the same buffer. After fixation, cells were dehydrated with ethanol (25%, 50%, 70%, 90%, and 100%) and embedded in Spurr resin. Ultra-thin sections (60 nm) were deposited on a 200-mesh copper grid and observed with a Zeiss Auriga Scanning Electron Microscope (Zeiss, Carl Zeiss Microscopy GmbH, Jena, Germany) equipped with the STEM module operating at 20 kV. Images were acquired at identical magnification and exposure settings for all experimental conditions. Regions of interest (ROIs) corresponding to heterochromatin areas were manually defined based on electron density as previously described [41]. Only foci (i) displaying a mean gray intensity at least two-fold higher than the surrounding nucleoplasmic area, and (ii) with an area greater than 10 µm^2^ were considered. Foci were analyzed using the Analyze Particles function in ImageJ (version 1.53—National Institutes of Health, Bethesda, MD, USA). The spatial position of each focus relative to the nuclear envelope was assessed by measuring the shortest distance between the focus and the nearest nuclear envelope using the Measure tool in ImageJ (version 1.53—National Institutes of Health, Bethesda, MD, USA). The number of analyzed nuclei for each condition is indicated in the corresponding figure legend.

### 2.5. Immunofluorescence

Cells were plated on coverslip and fixed after 48 h with 3.7% Formaldehyde (Merck, Darmstadt, Germany) for 10 min at RT. Samples were then permeabilized with 0.1% TRITON X-100 for 5 min at RT, blocked with 3% BSA and incubated sequentially with primary antibodies in 1% BSA for 1 h at RT and secondary antibodies in 0.025% TWEEN-20/PBS for 45 min at RT. The following primary antibodies were used: rabbit anti TRF1 (from de Lange T, Rockefeller University, New York, NY, USA [42]), mouse monoclonal anti ƔH2AX (Upstate Biotechnology, Lake Placid, NY, USA, JBW301), rabbit anti 53BP1 (Novus Biologicals, Centennial, CO, USA, NB100-304), and rabbit anti-CHMP7 (Proteintech, Rosemont, IL, USA, 16424-1 AP). Anti-rabbit Alexa Fluor 555 (A21430) and anti-mouse Alexa Fluor 488 (A21121) were used as secondary antibodies. DNA was stained with DAPI and slides were mounted with VECTASHIELD H-1000 (Vector Laboratories, Newark, CA, USA).

Immunofluorescence slides were examined with a Nikon Eclipse Ti2 (Nikon Instruments, Tokyo, Japan) epifluorescence microscope, or with the Zeiss LSM 800 (Carl Zeiss Microscopy GmbH, Jena, Germany) confocal microscope, or with a ZEISS LSM 900 (Carl Zeiss Microscopy GmbH, Jena, Germany) using a 100× objective (NA = 1.4, oil immersion) and Z-step of 0.5 μm. NIS—Elements and Zen 2.1 system software were, respectively, used for image acquisition. Identical acquisition settings for all experimental conditions were applied. Images were converted into maximum intensity projections prior to analysis, image contrast was adjusted uniformly across all samples using fixed display settings (minimum = 5, maximum = 120). Images were analyzed with Image J(version 1.53)/Fiji (version 2.9—National Institutes of Health, Bethesda, MD, USA) and Imaris (version 11.0.0—Bitplane, Oxford Instruments, Zurich, Switzerland) software. Three-dimensional volume and isosurface reconstructions of individual three-dimensional datasets were obtained with Imaris. Images were exported to Photoshop and Illustrator (version 27.2.0—Adobe, San Jose, CA, USA) for assembly.

Nuclear foci corresponding to γH2AX or 53BP1 signals were detected using the Find Maxima function in FiJi (version 2.9). Only nuclei displaying foci with a prominence value greater than 60 were included in the analysis. The same prominence threshold and analysis parameters were applied to all images and experimental conditions. Foci counts were obtained on a per-nucleus basis; the number of analyzed nuclei for each analysis is indicated in the corresponding figure legend.

The analysis of lobular nuclear protrusions relative to the rescue experiments was performed on HeLa cells fixed with 4% Methanol free Formaldehyde (Thermo Scientific, Rockford, IL, USA 28908) for 5 min at 37 °C. DNA was stained with DAPI, and slides were mounted with VECTASHIELD H-100 (Vector Laboratories, Newark, CA, USA). Images were acquired with a Nikon Eclipse Ti2 (Nikon Instruments, Tokyo, Japan) epifluorescence microscope. Maximum intensity projections were obtained with Imaris (version 11.0.0—Bitplane, Oxford Instruments, Zurich, Switzerland), and lobular nuclear protrusions were scored manually and counted with Photoshop. The number of analyzed nuclei for each condition is indicated in the corresponding figure legends.

The co-localization analysis of telomers and CHMP7 was performed on YFP-TRF1 HeLa. Cells were stained for CHMP7, images were then acquired with a Nikon Eclipse Ti2 epifluorescence microscope and denoised with Denoise.ai plug with NIS-Elements software (version 6.10.01—Nikon (Nikon Instruments, Tokyo, Japan)). Images were processed with Imaris (version 11.0.0—Bitplane, Oxford Instruments, Zurich, Switzerland) to obtain maximum intensity projections and exported to Photoshop (version 27.2.0—Adobe, San Jose, CA, USA) for the subsequent analysis. TRF1 foci and CHMP7 foci were manually counted on separate channels. Co-localization foci were manually counted on merged images. White foci in merged magenta (CHMP7) and green (YFP-TRF1) corresponding to overlapping signals from both channels were considered as co-localized foci. The number of analyzed nuclei for each condition is indicated in the corresponding figure legends.

Spatial intranuclear distribution of telomeres was analyzed following the approach described in [17]. Analyses were performed on asynchronous control (ctr) and CHMP7-reduced (shCHMP7) HeLa cells at passage 3 after puromycin selection. Immunofluorescence images of TRF1 were acquired and processed as maximum intensity projections prior to analysis. Nuclei were identified based on the DAPI signal, and their outlines were automatically defined in Image J(version 1.53)/Fiji (version 2.9—National Institutes of Health, Bethesda, MD, USA). Fit Ellipse function was used to determine the nuclear centroid and the lengths of the major and minor axes. These parameters were used to generate concentric elliptical regions of interest (ROIs) for radial position analysis. Three nuclear zones (Zone I, Zone II, and Zone III) were defined as previously described in [17]. Briefly the boundaries between zones were generated by scaling the major and minor axes of the fitted nuclear ellipse by factors of 0.577 and 0.816. Zone I corresponded to the peripheral nuclear region, Zone II to the intermediate region, and Zone III to the central nuclear region. TRF1-positive telomeric foci were identified using the Find Maxima function in FIJI, with a prominence threshold greater than 15. The same detection parameters were applied to all images and experimental conditions. Concentric regions of interest (ROIs) corresponding to the different nuclear zones were combined using XOR operations, allowing assignment of individual foci to a specific nuclear zone. The number of TRF1 foci in each zone was quantified on a per-nucleus basis. The number of analyzed nuclei for each condition is indicated in the corresponding figure legends.

### 2.6. FISH

FISH was carried out as previously described [43], and the telomeric probe was obtained by PCR as described by [44]. PCR products were sonicated to obtain 500–2000 bp fragments. After the hybridization reaction, the slides were washed 3 times in SSC4X-0.1% TWEEN-20, air-dried, and mounted in DAPI-Vectashield (Vector Laboratories, Newark, CA, USA).

Slides were examined with a Nikon Eclipse Ti2 epifluorescence microscope, or with the Zeiss LSM 800 confocal microscope, or with a ZEISS LSM 900 using a 100× objective (NA = 1.4, oil immersion) and Z-step of 0.5 μm. NIS—Elements and Zen 2.1 system software were, respectively, used for image acquisition. Images were acquired using identical acquisition parameters, including exposure time and gain, for all experimental conditions. Z-stack images were converted into maximum intensity projections prior to analysis. For each metaphase chromosome, telomeric signals present at both chromosomal ends were analyzed in terms of number, size, and relative spatial organization. Each chromosomal end was classified as either normal or aberrant based on established criteria reported in the literature [45,46,47,48,49]. Classification was performed consistently across all samples using the same criteria and analysis parameters. The number of analyzed chromosomes and ends for each sample is indicated in the corresponding figure legend. Images were analyzed and elaborated with Image J/Fiji (National Institutes of Health, Bethesda, MD, USA) and Imaris (version 11.0.0—Bitplane, Oxford Instruments, Zurich, Switzerland) software. Three-dimensional volume and isosurface reconstructions of individual three-dimensional datasets were obtained with Imaris. Images were then exported to Photoshop and Illustrator (version 27.2.0—Adobe, San Jose, CA, USA) for assembly.

### 2.7. Telomere Length Analysis

Genomic DNA was extracted using NucleoSpin Tissue Mini kit for DNA from cells and tissue (Macherey-Nagel GmbH & Co. KG, Düren, Germany) according to manufacturer’s instructions from control (ctr) and Chmp7-reduced (shChmp7) p53ko mouse embryonic fibroblasts at passage 3 post puromycin selection. Telomere length was evaluated using Absolute Mouse Telomere Length Quantification QPCR Assay Kit (ScienCell Research Laboratories, Carlsbad, CA, USA) according to manufacturer’s instructions. In brief, 50 ng of genomic DNA for each sample were amplified starting from telomere-specific or single-copy gene primers. QPCR reactions were performed in triplicate for each sample and the protocol used for amplification consisted of an initial denaturation step at 95 °C for 10 min, followed by 35 cycles of denaturation at 95 °C for 15 s, annealing at 52–58 °C for 30 s, and extension at 72 °C for 30 s. Data were analyzed as described [50] by normalizing telomere amplification to the single-copy gene and calculating mean telomere length, which provides an estimate of overall telomere status in the sample.

### 2.8. Statistics

Prism 10 software (GraphPad Software, Boston, MA, USA) was used to perform statistical analysis. The Kolmogorov–Smirnov or the Shapiro–Wilk test was used to assess the normality distribution of the data. For normally distributed data, unpaired Student’s *t*-test or Welch’s *t*-test was used to test for statistical significance between two groups. One-way ANOVA followed by Tukey’s post hoc test was used for comparisons among more than two groups. The Mann–Whitney test was performed on non-parametric data. One-way ANOVA Kruskal–Wallis followed by Dunn’s post hoc test was performed on non-parametric data for comparisons among more than two groups. *p*-values below 0.05 were considered significant and reported in the figures as * *p* < 0.05; ** *p* < 0.01; *** *p* < 0.001. *p*-values above 0.05 were considered not significant and were not reported in figures. Results are shown as mean ± SEM of at least three independent experiments.

## 3. Results

### 3.1. CHMP7 Reduction Induces Chromatin Alterations

Several pieces of evidence suggest a link between telomeres and the nuclear envelope. These are based on the analysis of the distribution of telomeres [17,18] and by studies focusing on cells with mutations in genes controlling the organization of the nuclear envelope such as A-type lamin mutations [19,20,21]. Considering the role of the ESCRT machinery in nuclear envelope reassembly and the pivotal activity in this context of CHMP7, we asked if CHMP7 was needed for telomere integrity.

**Figure 1:**
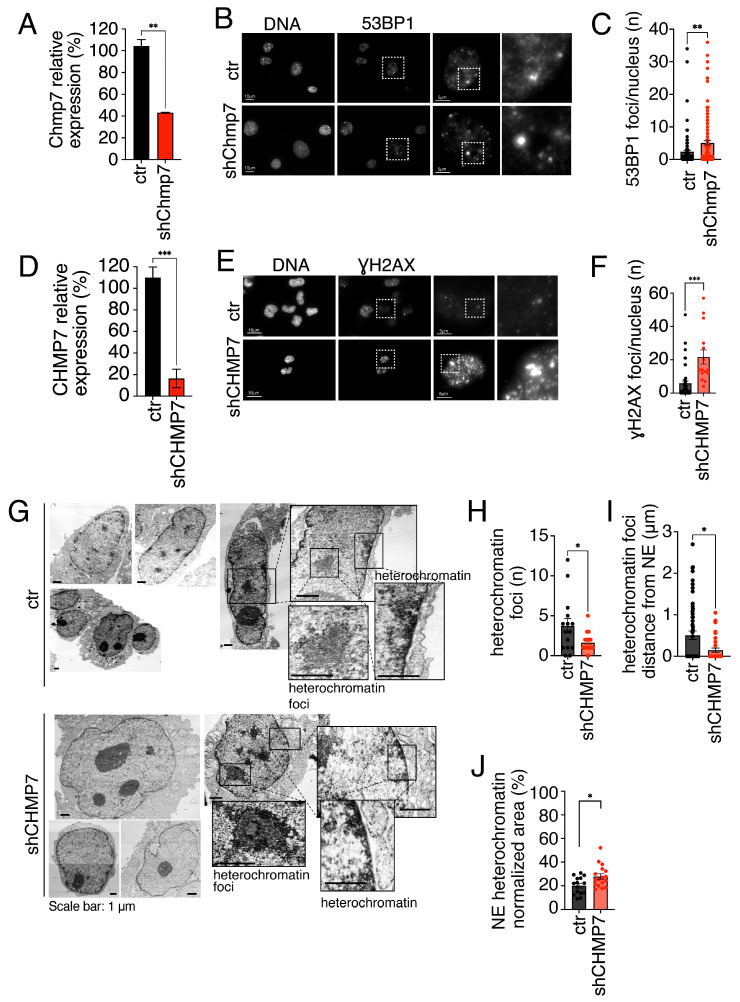
CHMP7 is needed for heterochromatin organization. (**A**) Gene expression quantification monitored by QPCR of control (ctr) and Chmp7-reduced (shChmp7) p53ko mouse embryonic fibroblasts. Statistics: unpaired two-tailed Student’s *t*-tests. (**B**,**C**) Representative immunofluorescence and relative foci quantification of ctr (*n* = 119) and shChmp7 (*n* = 132) p53ko mouse embryonic fibroblasts stained for 53BP1. Statistics: Kolmogorov–Smirnov test and non-parametric Mann–Whitney test. Dashed box highlights magnified elements reported on the right panel. (**D**) Gene expression quantification monitored by QPCR of control (ctr) and CHMP7 (shCHMP7) reduced HeLa cells. Statistics: unpaired two-tailed Student’s *t*-tests. (**E**,**F**) Representative immunofluorescence and relative foci quantification of ctr (*n* = 46) and shCHMP7 (*n* = 15) HeLa cells stained for ƔH2AX. Statistics: Kolmogorov–Smirnov test and non-parametric Mann–Whitney test. Dashed box highlights magnified elements reported on the right panel (**G**) Representative TEM images of control (ctr, *n* = 15) and CHMP7-reduced (shCHMP7, *n* = 17) HeLa cells. (**H**–**J**) Quantifications of TEM images shown in (**G**). Statistics: Mean ± SEM is shown. * *p* < 0.05, ** *p* < 0.01, *** *p* < 0.001.

As a first step, we set up two cell systems with reduced expression of CHMP7. To this goal, we used lentivirus-mediated RNA interference based on two different hairpin sequences targeting, respectively, Chmp7 in p53ko mouse embryonic fibroblasts and CHMP7 in HeLa cells. p53ko mouse embryonic fibroblasts and HeLa cells were chosen since they are both reference models for the study of telomeres. Indeed, they allow the identification of telomeric aberrations upon gene mutations and are not proliferation blocked by the reduction in factors controlling cell division [49,51]. The reduction in CHMP7 in the two cell types was validated by QPCR (Figure 1A,D) and by Western blotting (Supplementary Figure S1A–D). To confirm that cells tolerated the reduction in gene expression, we monitored in Chmp7-reduced p53ko mouse embryonic fibroblasts the population doublings and the mitotic index. Data show that Chmp7 reduction does not alter the proliferation and the rate of division of the cells at the conditions tested (Supplementary Figure S1E,F).

To further characterize the cell system, we performed rescue experiments in CHMP7-reduced HeLa cells by transfecting a CHMP7-GFP expression vector. The presence of the recombinant CHMP7-GFP fusion protein was assessed by Western blotting (Supplementary Figure S1G–I). The analysis of lobular nuclear protrusions in CHMP7-reduced and CHMP7-rescued cells showed that, consistently with its known function at the nuclear envelope, CHMP7 reduction significantly increased the percentage of irregular nuclei, while this phenotypic defect was rescued by CHMP7 over-expression (Supplementary Figure S1J,K).

Having defined the cell settings, we next proceeded to evaluate by immunofluorescence the presence of foci positive for the DNA damage marker 53BP1 (Figure 1B,C). We observed that Chmp7 reduction induces an increase in DNA damage foci as compared to control cells. DNA damage induction by CHMP7 reduction was confirmed when we analyzed foci positive for the DNA damage marker ƔH2AX in HeLa cells reduced of CHMP7 (Figure 1D–F).

We next evaluated by Transmission Electron Microscopy (TEM) the distribution of heterochromatin in CHMP7-reduced cells as compared to controls (Figure 1G–J). This ultrastructural analysis of control and CHMP7-reduced HeLa cells showed that CHMP7 is needed for the correct enrichment of heterochromatin at the rim of interphase nuclei. Indeed, both the heterochromatin foci and their distance from the nuclear envelope were reduced in CHMP7-reduced cells (Figure 1H,I). Moreover, the heterochromatin of CHMP7-reduced cells was less condensed since the quantification of its area showed a mild but significant increase in CHMP7-reduced cells as compared to controls (Figure 1J).

Taken together our data show that CHMP7 is needed to prevent the induction of DNA damage foci and that its reduction affects heterochromatin organization.

### 3.2. CHMP7 Is Required for Telomere Integrity

**Figure 2:**
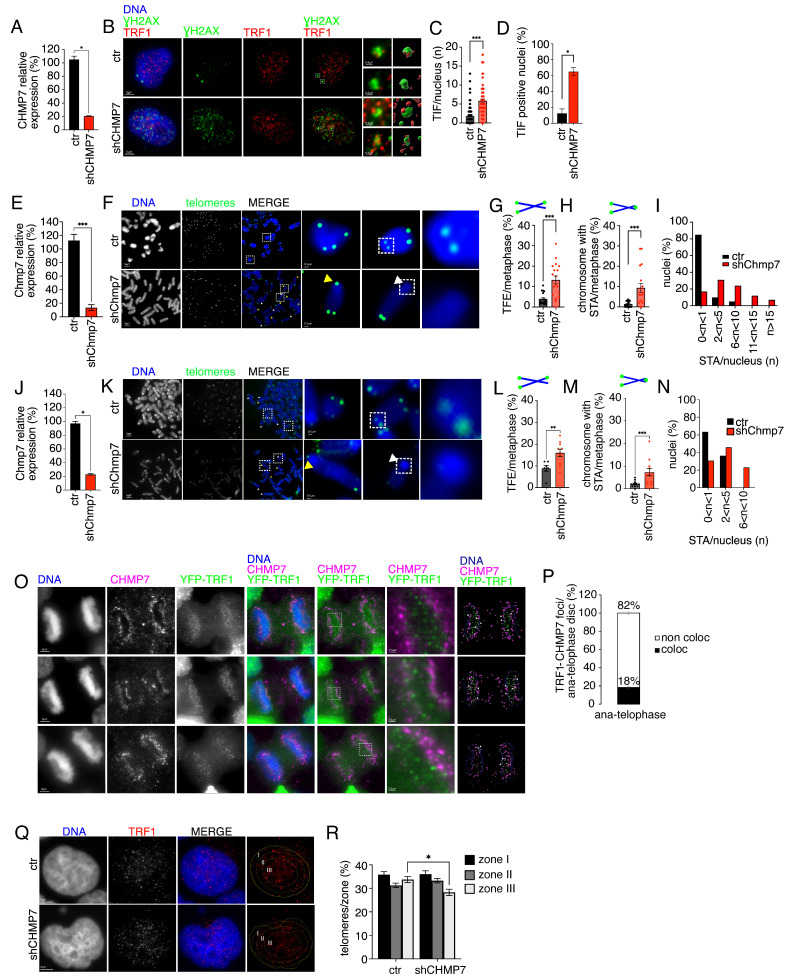
CHMP7 is needed for telomere integrity. (**A**) Gene expression quantification monitored by QPCR of control (ctr) and CHMP7-reduced (shCHMP7) HeLa cells. Statistics: unpaired two-tailed Student’s *t*-tests. (**B**–**D**) Representative immunofluorescence images of ctr (*n* = 145) and shCHMP7 (*n* = 61) HeLa cells stained for ƔH2AX (green) and TRF1 (red) and relative quantification of TIFs. Enlargements of ƔH2AX/TRF1 foci with isosurface reconstructions of individual 3D datasets are shown. Statistics: Kolmogorov–Smirnov test and non-parametric Mann–Whitney test. Dashed box highlights magnified elements reported on the right panel. (**C**); Shapiro–Wilk test and Welch’s *t*-test (**D**). (**E**) Gene expression quantification monitored by QPCR of control (ctr) and Chmp7-reduced (shChmp7) p53ko mouse embryonic fibroblasts derived from the mouse line described in [34]. Statistics: unpaired two-tailed Student’s *t*-tests. (**F**) Representative images of telomeric FISH on ctr and shChmp7 p53ko mouse embryonic fibroblasts. Yellow and white arrows indicate selected STAs and TFEs, respectively. Dashed box highlights magnified elements reported on the right panel. (**G**–**I**) Quantification of the frequency of telomeric aberrations from images as shown in (**F**) (ctr: n. of chromosomes = 840, n. of ends = 3360; shChmp7: n. of chromosomes = 720, n. of ends = 2880). Statistics: Kolmogorov–Smirnov test and non-parametric Mann–Whitney test. (**J**) Gene expression quantification monitored by QPCR of control (ctr) and Chmp7-reduced (shCHmp7) from p53ko mouse embryonic fibroblasts derived from the mouse line described in [33]. Statistics: unpaired two-tailed Student’s *t*-tests. (**K**) Representative images of telomeric FISH on ctr and shChmp7 p53ko mouse embryonic fibroblasts. Dashed box highlights magnified elements reported on the right panel. (**L**–**N**) Quantification of the frequency of telomeric aberrations from images as shown in (**K**) (ctr: n. of chromosomes = 440, n. of ends = 1760; shChmp7: n. of chromosomes = 480, n. of ends = 1920). Statistics: Kolmogorov–Smirnov test and non-parametric Mann–Whitney test (**L**) or Welch’s *t*-test (**M**). (**O**) Representative immunofluorescence images of YFP-TRF1 (green) HeLa cells stained for CHMP7 (magenta) and DAPI (blue). Enlargements, isosurface reconstructions of individual 3D datasets, and highlights (white arrow) of sites of association of telomeres and CHMP7 are shown. Dashed box highlights magnified elements reported on the right panel. (**P**) Quantification of YFP-TRF1 and CHMP7 co-localizing foci (n. of TRF1 foci = 987, n. of CHMP7 foci = 1940, n. of co-localizing foci = 181, n. of chromatin disks = 26). (**Q**,**R**) Representative images and signal distribution of TRF1/telomeres signals (red) in control (ctr: n. of nuclei = 28, n. of TRF1/telomeres = 2612) and CHMP7-reduced (shCHMP7: n. of nuclei = 26, n. of TRF1/telomeres = 2724) HeLa nuclei stained with DAPI. Statistics: Kolmogorov–Smirnov test and One-way ANOVA followed by Tukey’s post hoc test. Mean ± SEM is shown. * *p* < 0.05, ** *p* < 0.01, *** *p* < 0.001. I, II, III in (**Q**) highlight nuclear zones used for the quantification in (**R**).

Having assessed that CHMP7 reduction induces DNA damage foci and heterochromatin alterations, we next evaluated whether DNA damage foci were present specifically at telomeres. We thus monitored CHMP7-reduced HeLa cells for the presence of telomere-induced foci (TIFs) [51]. We analyzed, in particular, the co-localization of ƔH2AX and TRF1 in 3D volume and isosurface reconstructions of individual 3D datasets. The quantification of immunofluorescence images shows the significant increase both in terms of TIFs per nucleus and of TIF-positive nuclei in CHMP7-reduced cells as compared to controls (Figure 2A–D).

Considering the presence of DNA damage foci at telomeres and the alterations of heterochromatin in CHMP7-reduced cells, we hypothesized that CHMP7 reduction would drive alterations of the cytological organization of telomeres. To address this aspect, we analyzed telomeric aberrations by telomeric FISH (Fluorescence In Situ Hybridization). This technique is performed on metaphase spreads and allows not only to quantify the number of telomeric aberrations, but also to define the type of aberration. Namely, it allows to differentiate the presence of telomeric free ends (TFEs) and of sister telomere associations (STAs). We used p53ko mouse embryonic fibroblasts reduced of Chmp7 and monitored the presence of STAs and TFEs. The data show that the reduction in Chmp7 induces an increase in both these aberration types as compared to controls. Moreover, we observed that Chmp7-reduced metaphase spreads most frequently displayed 2 to 10 STAs per metaphase (Figure 2E–I). To extend the significance of the analysis we performed telomeric analysis not only in HeLa cells and in a batch of mouse embryonic fibroblasts derived from the mouse line described in [34], but also on a second batch of mouse embryonic fibroblasts that were derived from a different mouse line [33]. Telomeric FISH in these latter samples confirmed the impact of Chmp7 reduction on telomere integrity (Figure 2J–N).

Given the alterations of telomeres observed upon CHMP7 reduction, we next evaluated the co-localization of telomeres and CHMP7 by immunofluorescence. We focused on the ana-telophase stage during which CHMP7 foci are formed at the periphery of chromatin disks [29]. Imaging data of YFP-TRF1 HeLa cells show that 18% of the foci display co-localizing signals for TRF1 and CHMP7 (Figure 2O,P). To further address the link between CHMP7 and telomeres, we evaluated the distribution of telomeres in CHMP7-reduced HeLa cells. The data show an altered distribution of TRF1/telomeres upon CHMP7 reduction (Figure 2Q,R).

Finally, we performed the quantification of average telomere length by QPCR. Consistent with the presence of different types of telomeric aberrations which did not all converge into a telomere-loss phenotype (Figure 2E–N), we observed that the average telomere length upon CHMP7 reduction was comparable to that of control samples (Supplementary Figure S2).

Collectively these data show that CHMP7 is important for telomere maintenance both in human and in mouse cells, since its absence triggers telomeric damage, telomeric aberrations, and telomere spatial intranuclear alterations.

### 3.3. Telomere Integrity Is Affected by Multiple ESCRT Factors

**Figure 3:**
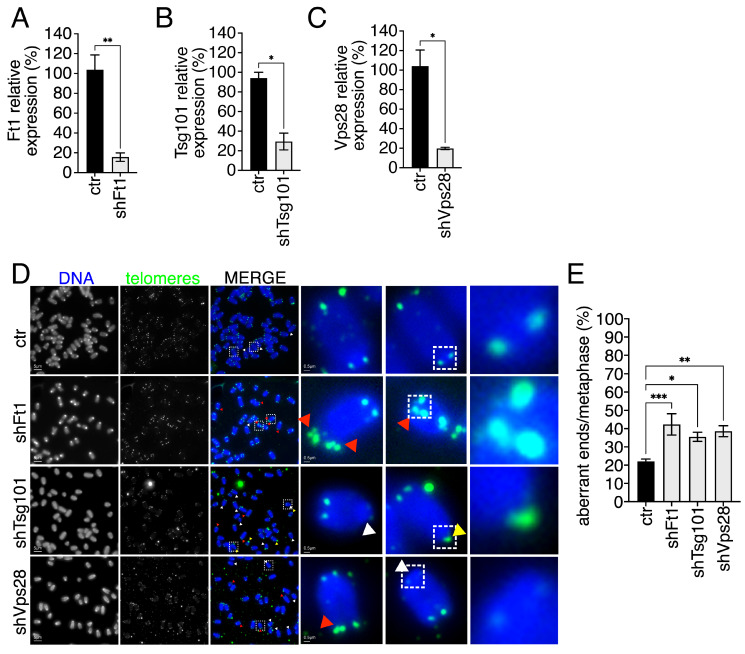
ESCRTs type I and AKTIP/Ft1 affect telomere integrity. (**A**–**C**) Gene expression quantification monitored by QPCR of control (ctr) and AKTIP/Ft1 (shFt1)-, Tsg101 (shTsg101)-, Vps28 (shVps28)-reduced p53ko mouse embryonic fibroblasts. Statistics: unpaired two-tailed Student’s *t*-tests. (**D**,**E**) Representative telomeric FISH images (**D**) and relative quantification (**E**) of telomeric aberrations of ctr (n. of chromosomes = 1120, n. of ends = 4480), shFt1 (n. of chromosomes = 760, n. of ends = 3040), shTsg101 (n. of chromosomes = 520, n. of ends = 2080), and shVps28 (n. of chromosomes = 680, n. of ends = 2720) p53ko mouse embryonic fibroblasts. Yellow, white, and red arrows indicate selected STAs, TFEs, and MTSs, respectively. Dashed box highlights magnified elements reported on the right panel. Statistics: Kolmogorov–Smirnov test and One-way ANOVA followed by Tukey’s post hoc test. Mean ± SEM is shown. * *p* < 0.05, ** *p* < 0.01, *** *p* < 0.001.

Given the multimeric nature of the ESCRT machinery, we asked whether other ESCRTs or ESCRT-associated factors would generate telomeric defects. We started from AKTIP/Ft1 and from the ESCRTs type I VPS28 and TSG101. AKTIP/Ft1 has extensive similarity with the ESCRT type I TSG101 and interacts with the ESCRT type I VPS28 [52]. The reduction in AKTIP/Ft1 was previously associated with telomere fragility [43,53]. We reduced p53ko mouse embryonic fibroblasts of AKTIP/Ft1, Tsg101, or Vps28 and monitored the reduction in the expression of each factor by QPCR (Figure 3A–C). Next, by telomeric FISH, we evaluated the presence of telomeric aberrations. Consistent with previous reports [43], data showed a significant impact of AKTIP/Ft1 reduction on the level of telomeric aberrations. Moreover, we observed a mild but statistically significant telomeric aberration induction also in Tsg101- and Vps28-reduced cells (Figure 3D,E).

**Figure 4:**
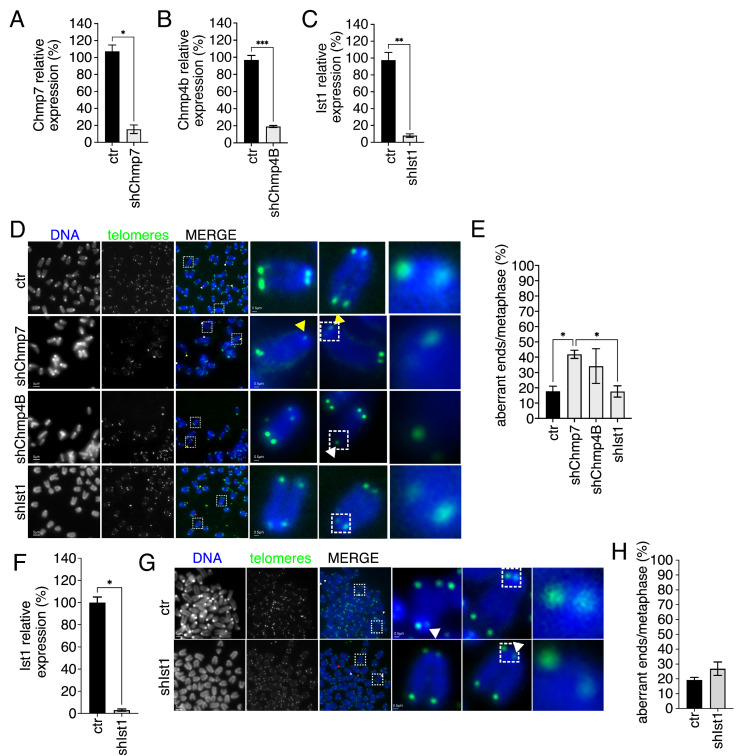
ESCRT type III CHMP4B affects telomere integrity. (**A**–**C**) Gene expression quantification monitored by QPCR of control (ctr) and Chmp7 (shChmp7)-, Chmp4B (shChmp4B)-, Ist1 (shIst1)-reduced p53ko mouse embryonic fibroblasts derived from the mouse line described in [34]). Statistics: unpaired two-tailed Student’s *t*-tests. (**D**,**E**) Representative images of telomeric FISH (**D**) and relative quantification (**E**) of ctr (n. of chromosomes = 320, n. of ends = 1280), shChmp7 (n. of chromosomes = 240, n. of ends = 960), shChmp4B (n. of chromosomes = 240, n. of ends = 960), and shIst1 (n. of chromosomes = 320, n. of ends = 1280) p53ko mouse embryonic fibroblasts derived from the mouse line described in [34]. Statistics: Kolmogorov–Smirnov test and One-way ANOVA followed by Tukey’s post hoc test. Yellow and white arrows indicate selected sister telomere associations and telomere free ends, respectively. Dashed box highlights magnified elements reported on the right panel. (**F**) Gene expression quantification monitored by QPCR of control (ctr) and Ist1 (shIst1) from p53ko mouse embryonic fibroblasts derived from the mouse line described in [33]. Statistics: unpaired two-tailed Student’s *t*-tests. (**G**,**H**) Representative images of telomeric FISH (**G**) and relative quantification (**H**) of ctr (n. of chromosomes = 560, n. of ends = 2240) and shIst1 (n. of chromosomes = 360, n. of ends = 1440) from p53ko mouse embryonic fibroblasts derived from the mouse line described in [33]. Statistics: Kolmogorov–Smirnov and Welch’s *t*-test. Yellow and white arrows indicate selected sister telomere associations and telomere free ends, respectively. Results are shown as the mean ± SEM. * *p* < 0.05, ** *p* < 0.01, *** *p* < 0.001.

We next analyzed the impact of the reduction in two ESCRT type III on telomeric integrity. Namely, CHMP4B and IST1, which were previously defined for their involvement in nuclear envelope reassembly [28,30]. We monitored the reduction in each factor by QPCR and then performed telomeric FISH analyses (Figure 4A–C). We observed a mild but significant impact on telomere aberrations of Chmp4B reduction and none for Ist1 (Figure 4D,E). In parallel samples, significantly higher impact was observed upon Chmp7 reduction (Figure 4A–E). To confirm that Ist1 had no impact on telomeric aberrations we performed telomeric FISH also in a second batch of p53ko mouse embryonic fibroblasts. The quantification of telomeric aberrations confirmed the absence of impact of Ist1 reduction on telomere integrity at least at the experimental conditions applied (Figure 4F–H).

These data suggest that the ESCRT type III CHMP4B contributes to telomere integrity. On the other hand, IST1 was not linked to telomere maintenance at the tested experimental conditions.

### 3.4. CHMP7 Genetically Interacts with the ESCRT CHMP4B and with AKTIP/Ft1

**Figure 5:**
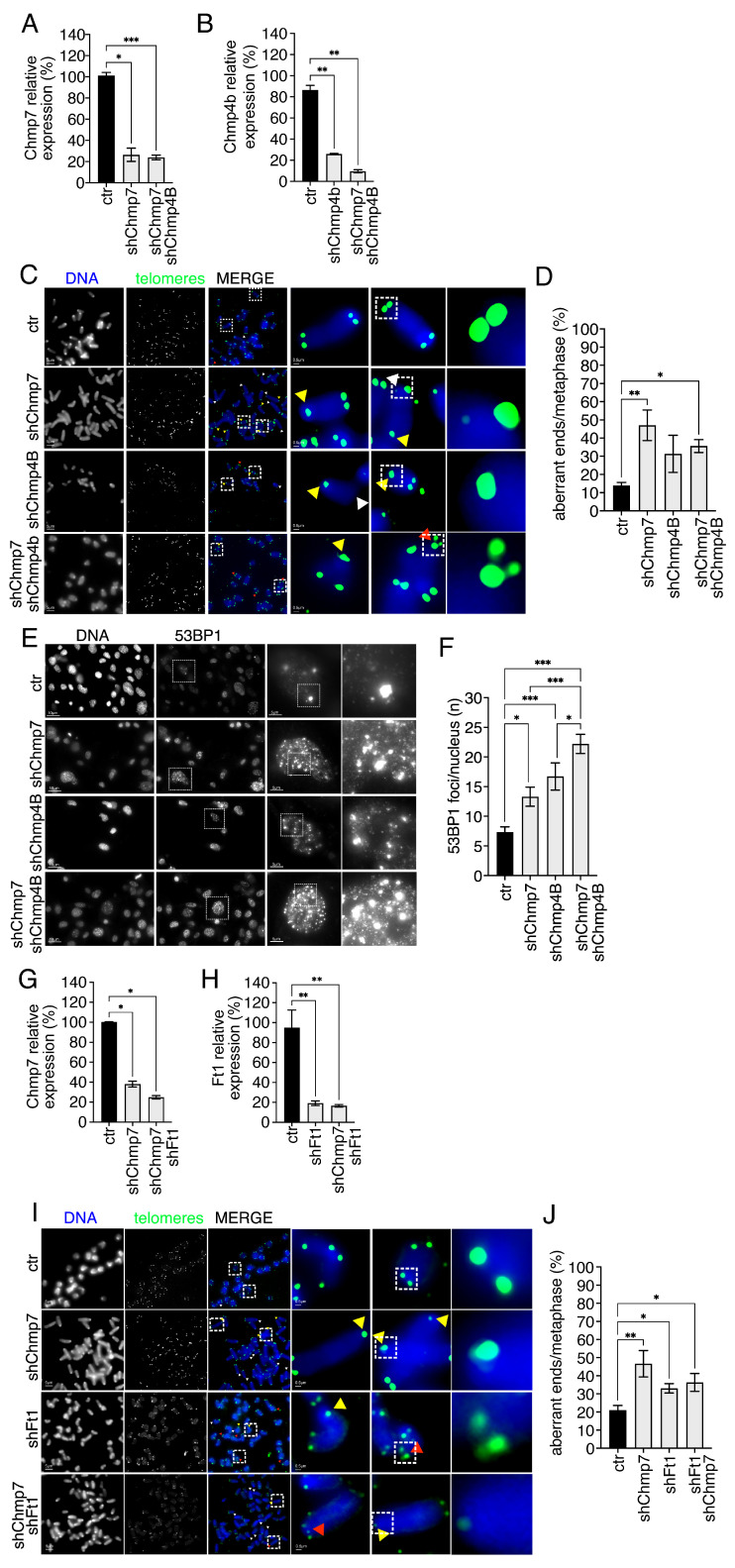
CHMP7 genetically interacts with AKTIP/FT1 and CHMP4B. (**A**,**B**) Gene expression quantification monitored by QPCR of control (ctr), Chmp7 (shChmp7), Chmp4B (shChmp4B), and double-reduced p53ko mouse embryonic fibroblasts. Statistics: unpaired two-tailed Student’s *t*-tests. (**C**,**D**) Representative images of telomeric FISH (**C**) and relative quantification (**D**) of ctr (n. of chromosomes = 320, n. of ends = 1280), shChmp7 (n. of chromosomes = 240, n. of ends = 960), shChmp4B (n. of chromosomes = 240, n. of ends = 960), and co-reduced (n. of chromosomes = 400, n. of ends = 1600) p53ko mouse embryonic fibroblasts. Statistics: Kolmogorov–Smirnov test and One-way ANOVA followed by Tukey’s post hoc test. Yellow, white, and red arrows indicate selected STAs, TFEs, and MTSs, respectively. Dashed box highlights magnified elements reported on the right panel. (**E**,**F**) Representative 53BP1 immunofluorescence images (**E**) and relative quantification (**F**) of 53BP1 foci per nucleus of ctr (*n* = 73), shChmp7 (*n* = 65), shChmp4B (*n* = 59) and co-reduced (*n* = 104) p53ko mouse embryonic fibroblasts. Statistics: Kolmogorov–Smirnov test and One-way ANOVA Kruskal–Wallis followed by Dunn’s post hoc test. Dashed box highlights magnified elements reported on the right panel. (**G**,**H**) Gene expression quantification monitored by QPCR of control (ctr), Chmp7 (shChmp7), AKTIP/Ft1 (shFt1), and double-reduced p53ko mouse embryonic fibroblasts. Statistics: unpaired two-tailed Student’s *t*-tests. (**I**,**J**) Representative images of telomeric FISH (**I**) and relative quantification (**J**) of ctr (n. of chromosomes = 360, n. of ends = 1440), shChmp7 (n. of chromosomes = 200, n. of ends = 800), shFt1 (n. of chromosomes = 760, n. of ends = 3040) or double-reduced (n. of chromosomes = 840, n. of ends = 3360) p53ko mouse embryonic fibroblasts. Statistics: Kolmogorov–Smirnov test and One-way ANOVA Kruskal–Wallis followed by Dunn’s post hoc test. Yellow, white, and red arrows indicate selected STAs, TFEs, and MTSs, respectively. Dashed box highlights magnified elements reported on the right panel. Results are shown as the mean ± SEM. * *p* < 0.05, ** *p* < 0.01, *** *p* < 0.001.

The ESCRT CHMP7 is required during nuclear envelope reassembly and co-localizes with CHMP4B at the two sides of the anaphase chromatin disks [29]. However, CHMP4B is required at other intracellular sites, as at the midbody during abscission [54]. Given the exclusive functional positioning of CHMP7 at the nuclear envelope as opposed to the implication of CHMP4B at multiple intracellular sites, we used genetic analyses to monitor whether they were acting convergently when driving telomeric aberrations. We performed telomeric FISH analyses on control, single, and doubly reduced Chmp7/Chmp4B p53ko mouse embryonic fibroblasts (Figure 5A–D). We used QPCR to monitor single-factor reduction (Figure 5A,B). The qualitative (Figure 5C) and quantitative analysis of aberrant telomeres (Figure 5D) showed that doubly reduced Chmp7/Chmp4B cells did not display additive alterations. Rather, the reduction in Chmp4B partly suppressed the impact of Chmp7 reduction on the phenotype. We next monitored DNA damage foci in double Chmp7/Chmp4B-reduced cells. We observed that the impact of double reduction was, in this case, additive (Figure 5E,F). A possible interpretation of these genetic interactions is that CHMP4B and CHMP7 reduction generates DNA damage foci via independent pathways, while they act on telomeres via a common route.

We next asked about the genetic interaction between the ESCRT-associated factor AKTIP/Ft1 and CHMP7 in the determination of the telomeric phenotype. To evaluate the interplay between AKTIP/Ft1 and CHMP7, we co-reduced their expression in p53ko mouse embryonic fibroblasts and quantified single-factor reduction by QPCR (Figure 5G,H). Next, we analyzed qualitatively and quantitatively the presence of telomeric aberrations. FISH analysis showed that the doubly reduced p53ko mouse embryonic fibroblasts did not display additive alterations, and that the reduction in AKTIP/Ft1 mildly suppressed the impact of Chmp7 reduction on the phenotype (Figure 5I,J). This genetic interaction possibly suggests that CHMP7 and AKTIP/Ft1 converge in the control of telomere integrity.

### 3.5. BAF1 and LEM2 Are Needed for CHMP7 Impact on Telomeres

**Figure 6:**
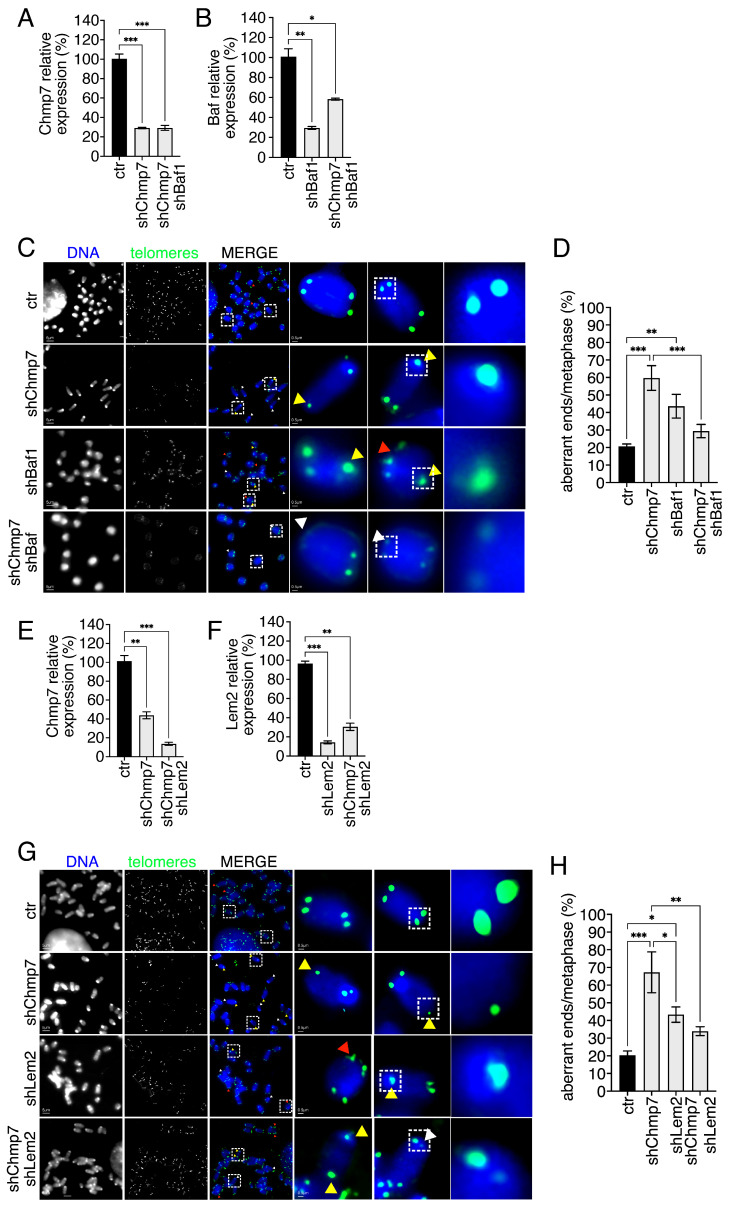
BAF1 and LEM2 together with CHMP7 are required for telomere integrity. (**A**,**B**) Gene expression quantification monitored by QPCR of control (ctr), Chmp7 (shChmp7), Baf1 (shBaf1), and double-reduced p53ko mouse embryonic fibroblasts. Statistics: unpaired two-tailed Student’s *t*-tests. (**C**,**D**) Representative images of telomeric FISH (**C**) and relative quantification (**D**) of ctr (n. of chromosomes = 600, n. of ends = 2400), shChmp7 (n. of chromosomes = 280, n. of ends = 1120), shBaf1 (n. of chromosomes = 400, n. of ends = 1600), and double-reduced (n. of chromosomes = 480, n. of ends = 1920) p53ko mouse embryonic fibroblasts. Statistics: Kolmogorov–Smirnov test and One-way ANOVA followed by Tukey’s post hoc test. Yellow, white, and red arrows indicate selected STAs, TFEs, and MTSs, respectively. Dashed box highlights magnified elements reported on the right panel. (**E**,**F**) Gene expression quantification monitored by QPCR of control (ctr), Chmp7 (shChmp7), Lem2 (shLem2), and double-reduced p53ko mouse embryonic fibroblasts. Statistics: unpaired two-tailed Student’s *t*-tests. (**G**,**H**) Representative images of telomeric FISH (**G**) and relative quantification (H) of ctr (n. of chromosomes = 280, n. of ends = 1120), shChmp7 (n. of chromosomes = 320, n. of ends = 1280), shLem2 (n. of chromosomes = 920, n. of ends = 3680), and double-reduced (n. of chromosomes = 520, n. of ends = 2080) p53ko mouse embryonic fibroblasts. Statistics: Kolmogorov–Smirnov test and One-way ANOVA followed by Tukey’s post hoc test. Yellow, white, and red arrows indicate selected STAs, TFEs, and MTSs, respectively. Dashed box highlights magnified elements reported on the right panel. Results are shown as the mean ± SEM. * *p* < 0.05, ** *p* < 0.01, *** *p* < 0.001.

Our data suggest that telomere integrity depends on the nuclear-envelope-associated ESCRT CHMP7. Considering the role of BAF1 in recruiting CHMP7 at the chromatin disks [55], we asked if CHMP7 role on telomeres was interlinked with BAF1. To this aim we singly or co-reduced Baf1/Chmp7 in p53ko mouse embryonic fibroblasts and validated single-factor reduction by QPCR (Figure 6A,B). We next analyzed by telomeric FISH the frequency of telomeric aberrations and observed a significant increase in the frequency of telomeric aberrations in Chmp7 and in Baf1-reduced cells (Figure 6C,D). In Chmp7/Baf1 co-reduced cells we did not observe an additive effect, but, rather, a robust suppressive effect of Baf1 over Chmp7 (Figure 6D). These data suggest that BAF1 and CHMP7 possibly act in a convergent pathway to ensure proper telomere maintenance. Moreover, it can be possibly hypothesized that BAF1acts upstream of CHMP7.

We next took into consideration the fact that it is known that during nuclear envelope reassembly BAF1 interacts with LEM2 to recruit CHMP7 [35,55]. Therefore, we asked whether LEM2 would have an impact on telomeres. To address this question, we singly and co-reduced Lem2/Chmp7 in p53ko mouse embryonic fibroblasts. We used QPCR to validate the reduction in single factors (Figure 6E,F). Next, by FISH, we could detect the significant increase in the frequency of telomeric aberrations both in single and co-reduced cells (Figure 6G,H). Interestingly, however, as in the case of BAF1, the frequency of aberrations in co-reduced cells was not additive with respect to singly reduced cells (both Lem2 and Chmp7). Rather, the double reduction was characterized by a suppressive effect of Lem2 on Chmp7. The fact that double depletion leads to a partial rescue of CHMP7 induced phenotype, albeit indirectly, argues against the interpretation that the gene-dependent telomeric defects are merely non-specific consequences of cellular stress.

Together, these data possibly suggest that BAF1 and LEM2 affect telomere integrity operating convergently, and, presumably, upstream to CHMP7.

### 3.6. CHMP7 and TNKS1 Affect Telomere Integrity by Independent Mechanisms

**Figure 7:**
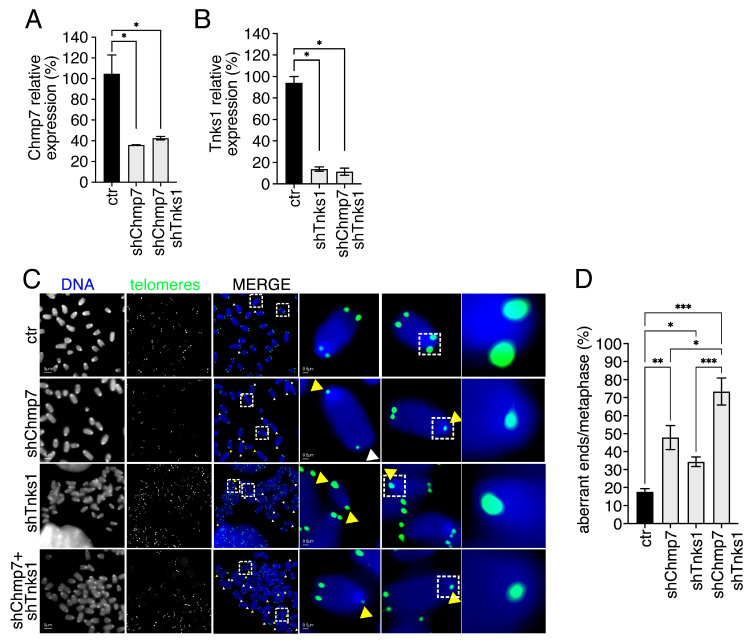
The impact of CHMP7 and TNKS1 on telomeres is additive. (**A**,**B**) Gene expression quantification monitored by QPCR of control (ctr), Chmp7 (shChmp7), Tnks1 (shTnks1), and double-reduced p53ko mouse embryonic fibroblasts. Statistics: unpaired two-tailed Student’s *t*-tests. (**C**,**D**) Representative images of telomeric FISH (**C**) and relative quantification (**D**) of ctr (n. of chromosomes = 480, n. of ends = 1920), shChmp7 (n. of chromosomes = 200, n. of ends = 800), shTnks1 (n. of chromosomes = 960, n. of ends = 3840), and double-reduced (n. of chromosomes = 480, n. of ends = 1920) p53ko mouse embryonic fibroblasts. Statistics: Kolmogorov–Smirnov test and One-way ANOVA followed by Tukey’s post hoc test. Yellow and white arrows indicate selected STAs and TFEs, respectively. Dashed box highlights magnified elements reported on the right panel. Results are shown as the mean ± SEM. * *p* < 0.05, ** *p* < 0.01, *** *p* < 0.001.

Telomere maintenance in mammalian cells depends on the shelterins and on telomeric-associated factors. One such factor is Tankyrase 1 (TNKS1), a regulator of the shelterin TRF1 associated with telomeric aberrations [3,4]. We asked if TNKS1 genetically interacted with CHMP7. To address this point, we produced singly and co-reduced Chmp7/Tnks1 p53ko mouse embryonic fibroblasts. We monitored by QPCR single-factor reduction (Figure 7A,B). We next analyzed by FISH telomeric aberration levels so we could observe the significant increase in telomeric aberrations in both Chmp7- and Tnks1-reduced cells as compared to controls (Figure 7C,D). In co-reduced cells we observed an additive effect with respect to the frequency of telomere aberrations in singly reduced cells.

**Figure 8:**
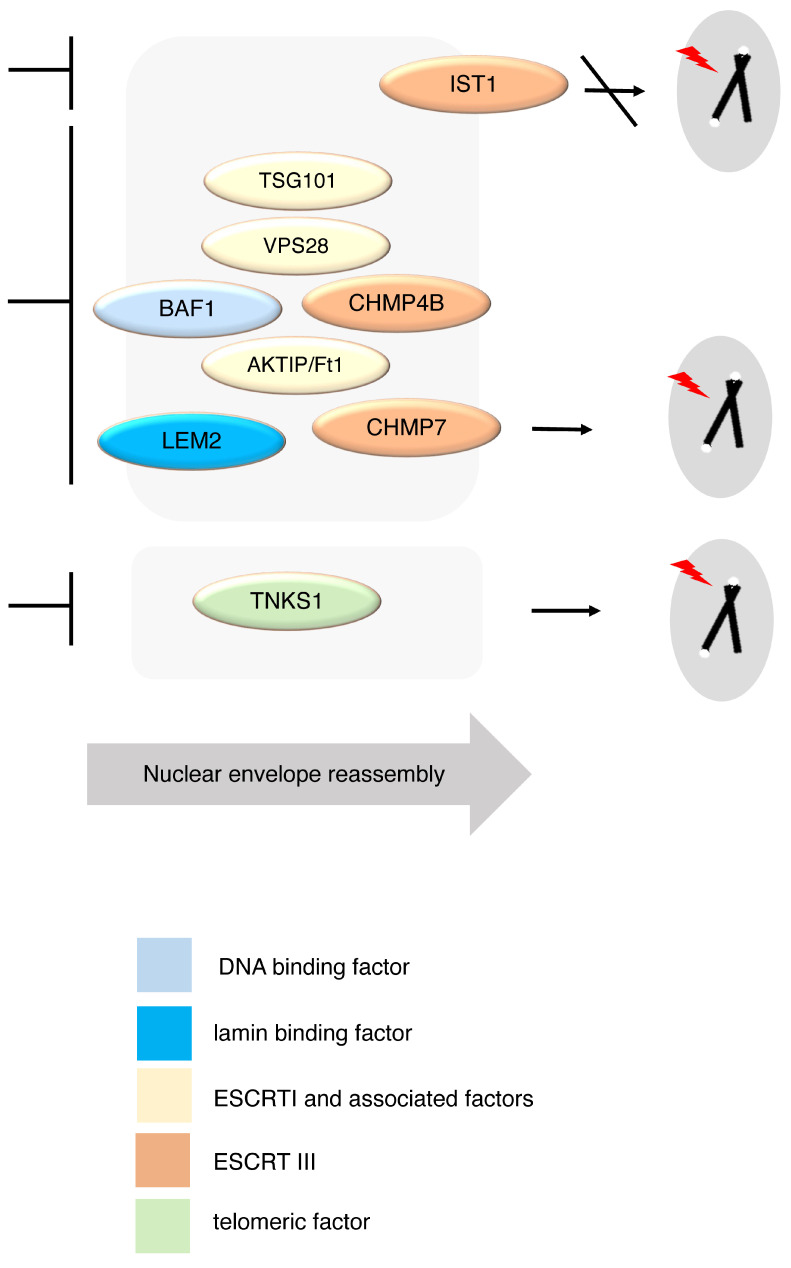
Schematic representation of the possible contextualization of CHMP7 contribution to telomere protection. The factors CHMP7, CHMP4B, LEM2, and BAF1, and AKTIP/Ft1 (playing a role at the nuclear envelope), and TSG101 and VPS28 (subcomponents of the ESCRT machinery) contribute to protecting chromosomes from telomeric aberrations. Presumably, they act convergently in a temporal sequence linked to the correct flux towards nuclear envelope reassembly. On the other hand, IST1 does not appear to be directly involved in telomere maintenance. The telomeric factor TNKS1 is confirmed in its importance for telomere integrity, but data suggest that it works via a CHMP7 independent pathway. Repression or promotion of the indicated processes is represented with t-shaped bars or black arrows, respectively.

These data taken together suggest that CHMP7, with other ESCRTs and ESCRT-associated factors, contributes to telomere maintenance. On the other hand, IST1 does not appear to be directly involved in the control of telomere maintenance. TNKS1 is confirmed in its importance for telomere integrity, consistently with its known function [3,4]. However, its role appears to be played in a different pathway as compared to ESCRTs and ESCRT-associated factors (Figure 8).

## 4. Discussion

Telomeres are tethered and controlled by the nuclear envelope. In yeast, the nuclear periphery organizes telomeres into the bouquet at the centrosome-spindle pole of the reforming nucleus [56]. In somatic mammalian cells, lamins, the main components of the nuclear envelope, control the topology and organization of telomeres [17,19,57], and contribute to repair of telomeres [19]. Moreover, LAP2alpha, a lamin-interacting protein, interacts with telomeres [23]. Crabbe and co-workers showed a SUN-dependent nuclear tethering of telomeres [17], which puts in relation not only telomeres with the nuclear envelope but also with the cytoskeleton [58].

In open mitosis, in the anaphase-telophase stage, components of the ESCRT machinery are recruited at the two sides of the chromatin disks by an aggregative process started by the lamina-associated factor LEM2 and by the chromatin binding factor BAF1 [35,55]. At this stage telomeres are enriched at the reforming nuclear envelope, namely they form clusters including the lamin-interacting factor LAP2alpha and BAF1 [17,18,23].

This convergent localization of telomeres, nuclear-envelope-associated factors and ESCRTs made us ask whether the nuclear-envelope-specific ESCRT factor CHMP7 would play a role in the maintenance of telomeres. To address this question, we used human and mouse CHMP7-reduced cells. We checked that CHMP7 expression was reduced at the RNA and at the protein level and that this reduction did not impinge on cell division. We also assessed that CHMP7 reduction generated the prototypical phenotypic defect of irregular nuclei as previously reported [59], and that the defects could be rescued by CHMP7 over-expression.

The first set of data indicating for a connection of CHMP7 with telomeres, which are mainly heterochromatic [60,61], was that heterochromatin was found altered in CHMP7-reduced cell. Moreover, we observed DNA damage activation upon CHMP7 reduction both in the HeLa cells and in mouse embryonic fibroblasts. Since studies on the ESCRT machinery have demonstrated that it limits DNA damage [62,63], our data are in line with these observations.

The second observation indicating for a connection of CHMP7 with telomeres was that DNA damage foci were detected at these structures and that their cytological organization was altered when CHMP7 expression was reduced. The most frequent telomere aberration types were STAs and TFEs. STAs occur when the telomeres of sister chromatids fail to separate properly, while TFEs are telomeric termini that have lost proper protective structure. Both types of aberrations are co-drivers in cancer and aging [64,65,66,67]. The experiments were performed in independent batches of human and mouse cells. However, to extend the comprehension of the impact of CHMP7 on telomeres, different cell settings, such as ALT+ cell lines or telomerase-negative primary fibroblasts, could be exploited in future studies.

The third set of data indicating for a connection of CHMP7 with telomeres was the observation of a partial co-localization of CHMP7 foci with TRF1/telomeric foci during anaphase, along with the altered TRF1/telomeric distribution in CHMP7-reduced interphase nuclei.

As a further step to dissect the connection between telomeres and the ESCRT machinery, we analyzed ESCRT factors other than CHMP7. We focused on canonical components of the machinery including ESCRTs type I and type III, and on factors associated with the ESCRT machinery and with the nuclear envelope, as the ESCRT-associated factor AKTIP/Ft1. Our data show that CHMP7 reduction exerts, as compared to all other tested factors, the highest effect on telomere integrity. However, a milder but significant impact is exerted as well by the reduction in ESCRTs type I TSG101 and VPS28, and by that of AKTIP/Ft1. While TSG101 and VPS28 have not been described yet at the nuclear envelope, AKTIP/Ft1 has been linked to telomere integrity and is enriched at the nuclear envelope in interphase cells [44,52,68]. The reduction in CHMP4B also had an impact on telomeres, but milder than that of CHMP7. Interestingly, IST1 did not appear to be essential for guaranteeing the integrity of telomeres. Consistent with these data, it has been shown in Saccharomyces cerevisiae, in a study focusing on the analysis of the impact of ESCRT depletion on telomeres, that vsp28 is involved, while Ist1 is not [69].

To go into the direction of deepening the interpretation of our data, we analyzed the genetic interactions between the different factors and CHMP7. We reasoned that, possibly, while non-additive effects would suggest the participation of the tested factors in a convergent pathway, additive ones would, on the other hand, indicate that the impact of each tested factor on telomere integrity would be exerted via independent routes. Using a co-depletion approach, we showed that CHMP7 exerts its action on telomeres acting convergently with the ESCRT type III CHMP4B. This is consistent with previous reports that indicate that these factors physically and functionally partner at the nuclear envelope. Interestingly, in double CHMP4B/CHMP7-reduced cells, differently from what we observed on telomeres, the activation of 53BP1 DNA damage foci was additive. This suggests that CHMP7 and CHMP4B impact on this latter aspect by independent routes, while on telomeres by a convergent one. A non-additive interaction was also observed for CHMP7 and AKTIP/Ft1. These data are compatible with the enrichment of both factors at the nuclear envelope and suggest that further investigation on the functional and biochemical interaction between AKTIP/Ft1 and the ESCRT components could be of interest to extend the knowledge on the mode of action of the machinery.

To further interpret the mode of action of CHMP7 in protecting telomere integrity, we extended our analyses to BAF1 and LEM2. Notably, it is upon aggregation of BAF1 and LEM2 at chromatin disks, during the anaphase/telophase stage, that CHMP7 and the other ESCRT factors are recruited for nuclear envelope reassembly [35,55]. We showed in this study that the reduction in BAF1 as that of LEM2 generated telomeric aberrations. Furthermore, we observed that BAF1 and—to a lower extent—LEM2 act as suppressors onto the CHMP7-dependent control of telomere integrity. These data together possibly suggest that CHMP7 acts on telomere maintenance together with BAF1 and with LEM2. Moreover, they are consistent with the knowledge that, with respect to CHMP7, BAF1 and LEM2 function upstream in the same pathway [35,55]. We cannot exclude, however, at this stage that the suppressive effects observed in double gene reductions could reflect compensatory mechanisms.

An internal conceptual control for our genetic approach and data interpretation derived from the analysis of the interaction of CHMP7 with TNKS1. TNKS1 is a telomere-specific factor, acting on the shelterin TRF1 [3,4]. Our genetic analyses showed that CHMP7 and TNKS1 are both required for telomere integrity. However, and differently from what was observed for the ESCRTs and ESCRT-associated factors, CHMP7 and TNKS1 presumably function in different pathways since the co-reduction generated additive effects on telomere aberrations.

## 5. Conclusions

Together, our data establish a contribution of CHMP7 in telomere integrity. To integrate this evidence into a broader framework, we propose a model in which telomere organization in mammalian cells critically depends on the proper ESCRT-dependent full assembly during nuclear envelope reformation and chromatin post-mitotic reorganization. CHMP7 emerges in this process, putatively acting in a shared pathway with other ESCRTs and ESCRT-associated factors, and downstream of the upstream recruiters BAF1 and LEM2. By contrast, its additive interactions with TNKS1 point to additional parallel routes.

Because defects in nuclear envelope dynamics and chromatin–nuclear membrane coupling underline several nuclear shape disorders and conditions of nuclear fragility, our results contribute to provide a further aspect linking ESCRT activity and these pathological states. In an applied perspective, defining the ESCRT dependent components that sustain telomere stability may help identify selective targets for modulating cell maintenance in diseases characterized by nuclear deformation or compromised envelope integrity, such as in aged cells and in cancer metastasis.

## Data Availability

The data presented in this study are available in article or Supplementary Materials.

## Supplementary Materials

The following supporting information can be downloaded at: https://www.mdpi.com/article/10.3390/cells15030256/s1, Figure S1: Characterization of CHMP7 reduced cells. (A,B) Western blotting hybridization with anti CHMP7 and anti actin antibodies on whole protein extracts from control (ctr) and Chmp7 reduced (shChmp7) HeLa cells and relative quantification (B). Statistics: Shapiro-Wilk test and non-parametric Mann–Whitney test. (C,D) Western blotting hybridization with anti-CHMP7 and anti-actin antibodies on whole protein extracts from control (ctr) and Chmp7 reduced (shChmp7) p53ko mouse embryonic fibroblasts, and relative quantification (D). Statistics: Shapiro-Wilk test and non-parametric Mann–Whitney test. Actin level was used as loading control. *: non specific band. Statistics: unpaired two-tailed Student’s *t*-tests. (E) Cumulative population doubling of control (ctr) and Chmp7-reduced (shChmp7) p53ko mouse embryonic fibroblasts. Statistics: Shapiro-Wilk test and Welch’s *t*-test. (F) Mitotic index of cells based on counting of DAPI-stained slides from control (ctr, *n* = 1282) and Chmp7-reduced (shChmp7, *n* = 1053) p53ko mouse embryonic fibroblasts. Statistics: Shapiro-Wilk test and non-parametric Mann–Whitney test. (G,I) Western blotting hybridization with anti-CHMP7 and anti-actin antibodies on whole protein extracts from control (ctr), CHMP7 reduced (shCHMP7) HeLa cells, and samples transfected with GFP-CHMP7 vector, and relative quantification (H,I). Statistics: Kolmogorov–Smirnov test and One-way ANOVA Kruskal-Wallis followed by Dunn’s post hoc test. (J,K) Lobular nuclear protrusions analysis based on DAPI stained slides from control (ctr, *n* = 93), CHMP7 reduced (shCHMP7, *n* = 123), CHMP7 reduced and control HeLa cells transfected with GFP–CHMP7 (shCHMP7–GFP–CHMP7, *n* = 88; ctr–GFP–CHMP7, *n* = 176), and relative quantification (K). Statistics: Kolmogorov–Smirnov test and One-way ANOVA Kruskal-Wallis followed by Dunn’s post hoc test. Yellow arrows indicate selected lobular nuclear protrusions. Dashed box highlights magnified elements reported on the right panel. Results are shown as the mean ± SEM. * *p* < 0.05, ** *p* < 0.01, *** *p* < 0.001. Figure S2: Telomere length analysis of Chmp7 reduced cells. QPCR showing average telomere length of control (ctr) and Chmp7 reduced (shChmp7) p53ko mouse embryonic fibroblasts. Statistics: Shapiro-Wilk test and non-parametric Mann–Whitney test.
