# Correction: Burla et al. Reduced CHMP7 Expression Compromises Telomere Integrity in Mammalian Cells. *Cells* 2026, *15*, 256

**DOI:** 10.3390/cells15100856

**Published:** 2026-05-08

**Authors:** Romina Burla, Mattia La Torre, Klizia Maccaroni, Stefano Tacconi, Marco Fidaleo, Luciana Dini, Isabella Saggio

**Affiliations:** 1 Department of Biology and Biotechnologies “Charles Darwin”, Sapienza University of Rome, 00185 Rome, Italy; romina.burla@uniroma1.it (R.B.); klizia.maccaroni@ifo.it (K.M.); stefano.tacconi@uniroma1.it (S.T.); marco.fidaleo@uniroma1.it (M.F.); luciana.dini@uniroma1.it (L.D.); 2 Institute of Molecular Biology and Pathology, CNR Rome, 00185 Rome, Italy

## Addition of an Author

In the original publication [1], Marco Fidaleo was not included as an author.

The corrected Author Contributions statement appears here. The authors state that the scientific conclusions are unaffected. This correction was approved by the Academic Editor. The original publication has also been updated.

**Author Contributions:** Conceptualization, I.S. and M.L.T.; methodology, I.S., R.B. and M.L.T.; software, M.L.T.; investigation, R.B.; data curation, I.S., R.B., M.L.T., K.M., L.D., S.T. and M.F.; writing—original draft, I.S. and R.B.; writing—review and editing, I.S. and M.L.T.; visualization, M.L.T.; supervision, I.S.; funding acquisition, I.S. All authors have read and agreed to the published version of the manuscript.

## References

[1] Burla R., La Torre M., Maccaroni K., Tacconi S., Fidaleo M., Dini L., Saggio I. (2026). Reduced CHMP7 Expression Compromises Telomere Integrity in Mammalian Cells. Cells.

