# Supplementary material for: Reduced CHMP7 Expression Compromises Telomere Integrity in Mammalian Cells
Source: Cells. 2026 Jan 28;15(3):256. doi: 10.3390/cells15030256 (PMC12896704; doi:10.3390/cells15030256)
Supplement: Supplementary file 1 [file cells-15-00256-s001.zip › cells-4042937-supplementary.pdf]

Supplementary figures

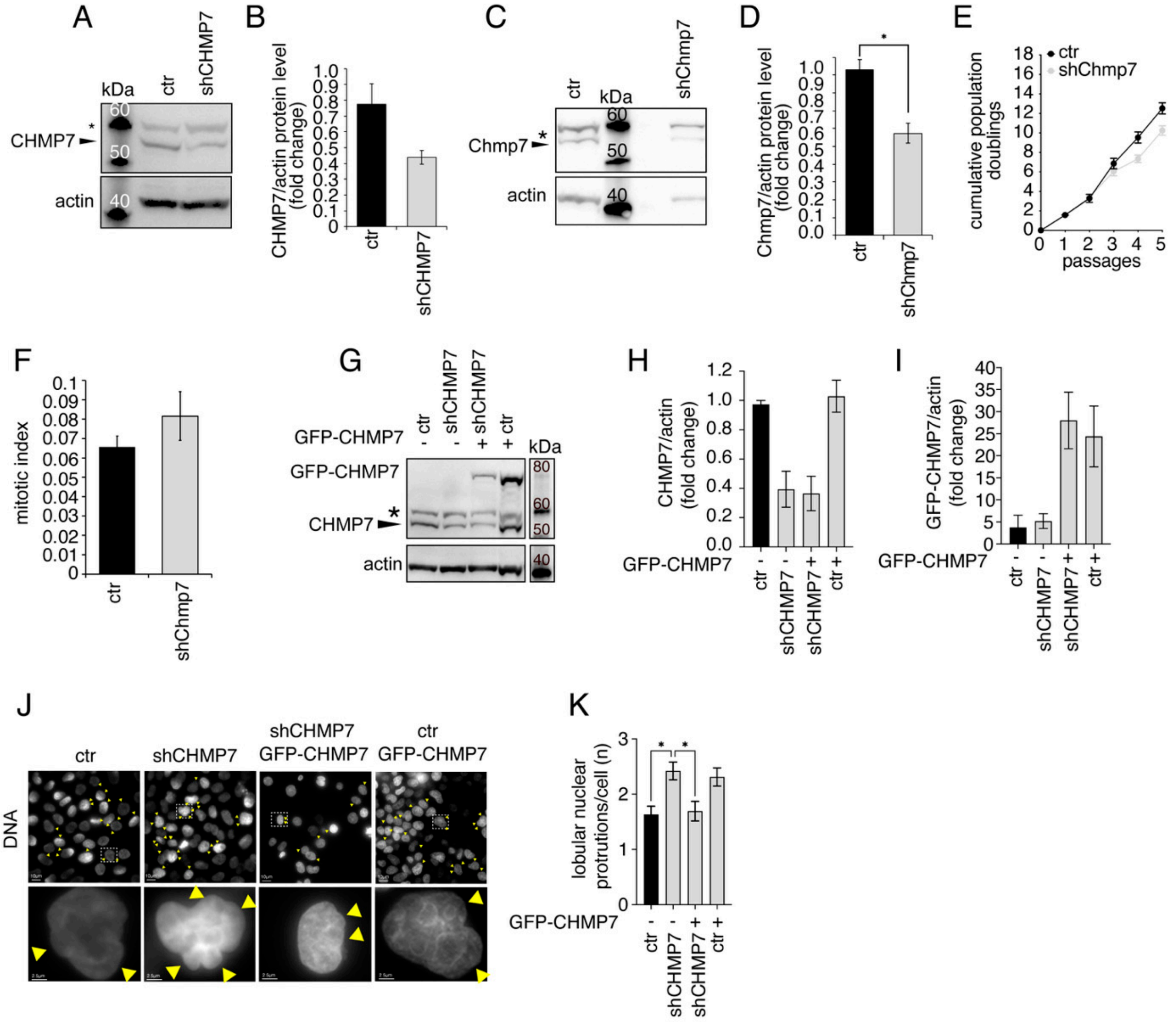

Supplementary Figure S1. Characterization of CHMP7 reduced cells. A-B Western blotting hybridization with anti CHMP7 and anti actin antibodies on whole protein extracts from control (ctr) and Chmp7 reduced (shChmp7) HeLa cells and relative quantification (B). Statistics: Shapiro-Wilk test and non-parametric Mann–Whitney test. **C-D** Western blotting hybridization with anti CHMP7 and anti actin antibodies on whole protein extracts from control (ctr) and Chmp7 reduced (shChmp7) p53ko mouse embryonic fibroblasts and relative quantification (D). Statistics: Shapiro-Wilk test and non-parametric Mann–Whitney test. Actin level was used as loading control. *: non specific band. Statistics: unpaired two-tailed Student's t-tests. **E** Cumulative population doubling of control (ctr) and Chmp7 reduced (shChmp7) p53ko mouse embryonic fibroblasts. Statistics: Shapiro-Wilk test and Welch's t-test. **F** Mitotic index of cells based on counting of DAPI stained slides from control (ctr, n=1282) and Chmp7 reduced (shChmp7, n=1053) p53ko mouse embryonic fibroblasts. Statistics: Shapiro-Wilk test and non-parametric Mann–Whitney test. **G-I** Western blotting hybridization with anti CHMP7 and anti actin antibodies on whole protein extracts from control (ctr), CHMP7 reduced (shCHMP7) HeLa cells, and samples transfected with GFP-CHMP7 vector, and relative quantification (H-I). Statistics: Kolmogorov–Smirnov test and One-way ANOVA Kruskal-Wallis followed by Dunn's post hoc test. **J-K** Lobular nuclear protrusions analysis based on DAPI stained slides from control (ctr, n = 93), CHMP7 reduced (shCHMP7, n = 123), CHMP7 reduced and control HeLa cells transfected with GFP–CHMP7 (shCHMP7–GFP–CHMP7, n = 88; ctr–GFP–CHMP7, n = 176), and relative quantification (K). Statistics: Kolmogorov–Smirnov test and One-way ANOVA Kruskal-Wallis followed by Dunn's post hoc test. Yellow arrows indicate selected lobular nuclear protrusions. Results are shown as the mean ± SEM. * p<0.05, **p<0.01, *** p<0.001.

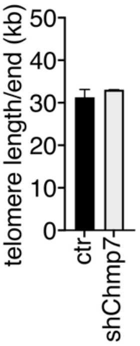

Supplementary Figure S2. Telomere length analysis of Chmp7 reduced cells. QPCR showing average telomere length of control (ctr) and Chmp7 reduced (shChmp7) p53ko mouse embryonic fibroblasts. Statistics: Shapiro-Wilk test and non-parametric Mann–Whitney test.
